# Association of stress with nutrition literacy, eating behavior, and physical activity: A cross-sectional study of university students in Bangladesh

**DOI:** 10.1371/journal.pone.0326269

**Published:** 2025-06-23

**Authors:** Towhid Hasan, Nibadita Majumder, Marjia Sultana, Md. Mahmudul Hasan Shohag, Nishat Subah Tithi

**Affiliations:** 1 Department of Food Technology and Nutrition Science, Noakhali Science and Technology University, Noakhali, Bangladesh.; Patuakhali Science and Technology University, BANGLADESH

## Abstract

**Background:**

Increased levels of stress among university students have an effect on their physical and mental well-being and may lower their learning outcomes and overall satisfaction. While lifestyle factors like nutrition literacy, eating behavior, and physical activity are known to influence stress, their combined association has not been explored among university students in Bangladesh.

**Objectives:**

This study intended to identify the level of perceived stress among university students in Bangladesh and explore its relationship with their nutrition literacy, healthy eating behavior, and physical activity.

**Methods:**

This cross-sectional study was conducted from May to June 2024 among 450 students of Noakhali Science and Technology University, Bangladesh. Data were collected through a structured questionnaire assessing socio-demographic characteristics, perceived stress (perceived stress scale [PSS]), nutrition literacy (nutrition literacy scale [NLS]), healthy eating behavior (healthy eating behavior scale [HEBS]) and physical activity (international physical activity questionnaire – short form [IPAQ-SF]).

**Results:**

Results indicated a moderate level of perceived stress among students, having a mean PSS score of 19.88 ± 4.67 out of 40 points. About half of the students had low nutrition literacy (57.3%) and poor healthy eating behavior (47.6%); however, almost all did sufficient physical activity (97.3%). Adjusted linear regression analysis revealed a significant negative association of perceived stress with nutrition literacy (*β* = –0.130, 95% confidence interval [CI]: –0.186, –0.092; P = 0.006) and healthy eating behavior (*β* = –0.139, 95% CI: –0.232, –0.028; P = 0.003) among university students. However, physical activity was not significantly associated with their perceived stress level (*β* = –0.039, 95% CI: –0.088, 0.010; P = 0.395).

**Conclusion:**

The present findings suggested that nutrition literacy and healthy eating behavior significantly impact perceived stress among university students, highlighting an urgent need for holistic health intervention in academic settings.

## Introduction

University age, an emerging adulthood period, is a unique part of human life where students pass through the most vital length of lifestyles and ride many complex events. As education progresses to the higher stage in university, the students face extra laborious activities like advanced syllabus, regular assignments and projects, dwelling in hostels, etc. Physical and psychological disturbances arise among the students when their adaptive competency does not work according to the requirements of the new environment of the university [1]. Psychological stress arises when external environmental demands exceed an individual's perceived ability to cope [2]. Research has suggested that mental stress is often linked to conditions such as depression, anxiety, and physical health issues, which may contribute to the development of cancers and cardiovascular diseases later in life [3]. People regularly experience stress when they understand they have limited capacity to cope with difficult situations effectively [2].

Various psychological and cognitive studies conducted in both developed and developing nations have reported a high incidence of stress, anxiety, and depression among students [3,4], resulting in an increased tendency to commit suicidal acts [2]. Previous research reported gender as an influential factor for stress, and females pronounced greater tiers of daily stressors than males [2]. Papier et al. [5] observed that half of the first-year university students at Australia experienced stress, where females suffered more than males, and that stress was observed to be linked to a lower intake of healthy foods. Students have a general tendency to choose unhealthy foods instead of nutritious foods. Choi [6] concluded that college students with high stress followed unhealthy eating behaviors, such as consuming ready-to-eat meals.

Nutrition literacy stems from the broader concept of health literacy, focusing on an individual's ability to use nutrition-specific knowledge to improve personal health through dietary choices [7]. Nutrition literacy is the capacity to access, comprehend, and interpret nutrition information, along with the skills required to make informed nutrition-related decisions [8]. Healthy eating habits and improved mental health in young people were observed to be positively associated with nutrition literacy [9]. The eating behavior and food intake of university students are often affected by multiple variables, such as, dietary restrictions, cooking abilities, knowledge and beliefs, peer influence and social expectations, campus environment and exam frequency, food preferences, access to cooking tools and facilities, and the cost of food [10]. Physiological stress may affect eating behavior, increasing or decreasing food consumption. It was observed that the consumption of unhealthy foods (i.e., processed foods, red meat, alcohol, etc.) increases while wholesome foods, including vegetables and fruits, decrease stress conditions [11].

Physical activity is characterized by any bodily movement that involves energy expenditure, including activities like walking, climbing stairs, or shopping [12]. Regular physical activity has been proven to offer numerous health advantages, such as improving body composition and lowering the risk of cardiovascular diseases. Regular physical activity also has an advantageous impact on mental health and emotional response, such as reminiscence, boosting self-esteem, reducing stress, and enhancing focus and engagement on a cognitive and social level [13]. The beneficial impacts of consistent physical activity are associated with the release of endorphins, which aid in mood regulation and improve the function of the prefrontal cortex, the brain region responsible for emotional control and decision-making [14]. Previous studies indicate that physical activity not solely promotes health via a direct discount of threat factors for significant diseases but also acts passively through stress-buffering consequences. Several studies supported the claim that people with excessive workout tiers exhibit fewer fitness problems if exposed to undue stress [15,16]. Engaging in physical activity can serve as a way to relieve stress and help individuals manage their emotions by allowing them to process and regulate their feelings [14].

Over the past decade, growing attention has been given to the causes of stress and its impact on the well-being of university students. The stress levels and mental health of these students are significant public health concerns, as they represent the future professional workforce. Students’ experiences at university have the potential to be fulfilling and productive. Empirical data suggests that being a student can occasionally become stressful [17]. Previous literature suggests that the status of students’ mental health is associated with their background characteristics [18]. Many universities in Bangladesh have recognized the importance of students’ mental health and well-being, yet their challenges often go unnoticed. Moreover, understanding the impact of nutrition literacy, physical activity, and eating behavior on stress is vital because these factors can help reduce stress-related health issues [19]. Proper nutrition literacy enables individuals to make healthier dietary choices, improving mood and resilience to stress [7,9]. In addition, sufficient physical activity is well-documented for its stress-relieving benefits [15,16], while healthy eating habits support mental and physical well-being [20]. By identifying how these behaviors influence stress levels, public health initiatives can better design programs that promote and support improved mental health of university students. Thus, this study aims to ascertain the level of perceived stress among university students in Bangladesh and to determine its association with their nutrition literacy, eating behavior, and physical activity. To the authors’ knowledge, this is the first study in Bangladesh assessing the association of stress with nutrition literacy, eating behavior, and physical activity.

## Methods

### Study design and participants

This is a cross-sectional study which was conducted among students of Noakhali Science and Technology University (NSTU) from 19/05/2024–02/06/2024 to examine the association of their stress with nutrition literacy, eating behavior, and physical activity. NSTU is a public university in the coastal terrain Noakhali of Bangladesh, with a land area of 101 acres (0.41 km^2^). Currently, it has more than 6000 students (both undergraduate and graduate) studying at various faculties. This university was chosen because of the convenience of data collection, in addition to limited funding and insufficient resources to expand the survey to other institutions across the country, and it has a diverse student population across multiple faculties. All currently enrolled students across various faculties were eligible, excluding those who were critically ill or declined participation. The required sample size was calculated from Cochran’s formula [21]:


$$Z^{2}pq/d^{2}$$

Where Z is 1.96 at a 95% confidence interval, p is the estimated proportion of the population (i.e., assuming 50% in this case), q is 1-p, and d is the margin of error at 5%. From the equation, 384 students would be necessary. To account for potential non-responses (assuming around 10%), a final sample of 450 students was targeted to ensure statistical confidence. A simple random sampling method was employed to select students, enhancing the representativeness of the sample by covering diverse faculties and academic levels, from undergraduate to postgraduate, across the university.

### Study instruments

A structured questionnaire was prepared first in English based on previously published literature. Two public health experts reviewed the draft questionnaire, and adjustments were made based on their feedback. To ensure clarity and avoid bias, the English version was translated into Bengali and then back-translated into English by two bilingual experts. The corrected version was then pilot-tested (from 13/05/2024–14/05/2024) on a group of 30 random students to assess the validity of the questionnaire and its efficiency. The results of pilot testing were shown in S1 Table. Necessary modifications were performed, depending on the responses, and finally employed for data collection purposes. The data and results of the pilot study were not included in the final analyses. The complete questionnaire is comprised of two major parts. The first part included the socio-demographic characteristics of the participants, and the second part assessed their perceived stress, nutrition literacy, healthy eating behavior, and physical activity level.

### Socio-demographic characteristics

The socio-demographic section included age, gender (male or female), accommodation (university hall, with family, with friends or in mess), academic status (first year, second year, third year, fourth year or masters), faculty of studying (science, engineering, business or arts), marital status (unmarried, currently married, divorced, separated or widowed), family residence (rural or urban), monthly family income, father's education (no formal schooling, primary, secondary or tertiary), father's occupation (service or self-employed), mother's education (no formal schooling, primary, secondary or tertiary), and mother's occupation (service or housewife).

### Perceived stress scale

Perceived Stress Scale (PSS), a self-reported measure, was used in this study to assess the extent to which students perceive situations in their university life as extremely stressful compared to their capacity to cope. It is a valid and reliable scale established as the most widely used measure of subjective stress globally [22]. PSS scale has three versions comprising, i.e., 14-items, 10-items, and 4-items, and among these, the 10-items (PSS-10) has shown better reliability and validity (Cronbach's α of 0.78 ± 0.91; test-retest reliability coefficients of 0.55 ± 0.80) compared to the other two versions [23–25]. The scale consists of six positively worded items (1, 2, 3, 6, 9, 10) and four negatively worded items (4, 5, 7, 8). Each item is rated on a five-point Likert scale, ranging from 0 (never) to 4 (very often). During the analysis, the scores of the negatively worded items were reversed. Consequently, the total scores observed were from 0 to 40, and the participants with higher scores were considered to have higher perceived stress levels. The reliability of the scale was verified using Cronbach’s coefficient alpha (α = 0.746).

### Nutrition literacy scale

A self-rated nutrition literacy (NLS) scale was adapted from Liao et al. [10] and Banna et. al. [26] which comprises five domains with eight items where two items for “obtain” (to estimate their potentiality of searching nutrition-related information), two for “understand” (to determine their nutritional knowledge), one for “analysis” (to assess their ability to differentiate nutrition-related knowledge in particular conditions), two for “appraise” (to evaluate their capability to judge and decide nutrition-related information for their own needs) and one for “apply” (to determine their ability to implement nutrition-related information to their daily life for better health and well-being). Their scores were evaluated using a 4-point Likert scale varying from 1 (very difficult) to 4 (very easy). All items from NLS were summed, and the total score was divided by the mean value. Scores below the mean were categorized as low nutrition literacy, while scores above the mean were categorized as high nutrition literacy. Cronbach’s coefficient alpha (α = 0.823) was used to assess the reliability of NLS.

### Healthy eating behavior scale

The healthy eating behavior scale (HEBS) used in this study was adapted from Banna et al. [27] to evaluate whether an individual follows the National Dietary Guidelines of Bangladesh. This guideline comprised advisory statements outlining principles and criteria for promoting overall health and well-being. It also recommends healthy cooking and preparation techniques to preserve micronutrients in food. The HEBS contained 17 items regarding people's eating habits and nutrition-related behaviors. Three options were available for each item (except 14 and 16). These were “regular” (every day/week),” “occasionally (≥3 days/week),” and “never.” The answers to question 14, “Has your body weight been measured?” were divided into “regular (≥1/month),” “occasionally (once in 2–3 months)” and “never.” The other question (Question 16) about the health check-up entitled “undertake clinical check-up?” had three possible answers: “regular” (once a year),” “occasionally” (once in 2–3 years),” and “never.” This study used a scoring system of 0 for “regular,” 1 for “occasionally,” and 2 for “never,” to evaluate participant’s healthy eating behavior. For simplicity of understanding and scoring, the score for the item “Eat foods containing excessive fats and oils/eating fast foods” was inverted during analysis. The total score ranged from 0 to 34, based on the summation of the scores of 17 items. The total score was then divided by the mean score and scores below the mean were categorized as poor healthy eating behavior, while scores above the mean were categorized as good healthy eating behavior. The HEBS showed an acceptable reliability, having Cronbach's α of 0.721.

### Physical activity

Physical activity was assessed using the International Physical Activity Questionnaire – Short Form (IPAQ-SF), covering the previous seven days [28]. This questionnaire pertained to 7 items, recording the activity of four intensity levels: 1) vigorous-intensity activity, such as heavy lifting, digging, aerobics, or fast bicycling, etc., 2) moderate-intensity activity, such as carrying light loads, bicycling at a regular pace, or doubles tennis, etc., 3) walking, and 4) sitting. The total physical activity of the participants was presented as the estimation of energy expenditure in metabolic equivalent tasks (MET)-minutes/week. Total METs (continuous score from the IPAQ-SF scoring protocol) were calculated as follows: (daily minutes of vigorous-intensity activity × days per week with vigorous-intensity activity × 8.0) + (daily minutes of moderate-intensity activity × days per week with moderate-intensity activity × 4.0) + (daily minutes of walking × days per week with walking × 3.3). Participants were classified as having sufficient physical activity when their total physical activity was observed to be ≥ 600 MET-minutes/week [28].

### Data collection

Ethical approval was taken from the Ethics Committee of Noakhali Science and Technology University, Bangladesh, (Reference no: NSTU/SCI/EC/2024/248) prior to data collection. The authors, with the help of some graduate students, approached the prospective participants via advertisements, emails, and class announcements. Informed consent was obtained by fully briefing participants on the study's objectives, procedures, potential benefits, and associated risks before participation. In accordance with regulations aimed at protecting human subjects, both oral and written consent were collected from students who agreed to join the study, ensuring that participation was entirely voluntary. Students were assured that they could withdraw from the study at any time without any consequences. The participants received guarantees about the privacy of their data and identities. To maintain confidentiality, all responses were anonymized, with personal identifiers removed from data records. Information was gathered through a face-to-face interview session with the participants. Approximately 15 minutes were required to complete the whole data collection process, using a printed copy of the final questionnaire along with “Bangladeshi Food Pyramid” (to understand the fourth and fifth items of the Nutrition Literacy Scale).

### Statistical analysis

This study assessed the validity and reliability of the scales using various statistical techniques. The study applied the following criteria to determine a scale's goodness of fit: the comparative fit index (CFI) and Tucker-Lewis index (TLI) of ≥0.95 [29], the root mean square error of approximation (RMSEA) of ≤0.06 [30], and the standardized root mean square residual (SRMR) of <0.08 [29]. The scale's fitness was also evaluated through the relative chi-square method, where a chi-square to degrees of freedom ratio (χ2/*df*) ≤2.00 indicated a good fit [31]. Additionally, Cronbach's α coefficients were used to assess the internal reliability of each scale.

The socio-demographic and perceived stress scores were presented by frequency, percentages, mean, and standard deviation (SD) where necessary. The Shapiro-Wilk test was performed for the continuous variables to determine the normality of the distribution. Pearson’s correlation was conducted to ascertain the relationship of perceived stress with nutrition literacy, eating behavior, and physical activity, while the association between the variables detailed was analyzed using the *t*-test. Additionally, a multiple linear regression analysis was performed in order to control the relationship between each variable. For the model, the assumptions of the multiple linear regression models (i.e., normality, homoscedasticity of variances, independence, and linearity of the residuals) were tested. In this study, both categorical and continuous measures of the variables (nutrition literacy, healthy eating behavior, and physical activity) were employed to provide a comprehensive understanding of their relationships with perceived stress. While the categorical presentation provides a simplified overview, the regression analysis employs continuous variables to yield more accurate and interpretable effect sizes. Scale and model fit analyses were conducted in SPSS AMOS (version 24.0) for Windows (IBM Co., Armonk, NY, USA), whereas all other analyses were done using SPSS (version 27.0) for Windows (IBM Co., Armonk, NY, USA). A P < 0.05 was set as statistical significance.

### Results

The socio-demographic profile of the study participants is presented in Table 1. More than half of the respondents were female, and the majority were 21–25 years old. Around three-fourths of the students lived in university residences, and most of them were undergraduates. More than one in two participants were from the science faculty, and most of them noticed being unmarried. A higher proportion of the students resided in urban areas, and about two-thirds reported their monthly family income was above 15000 Bangladeshi taka (BDT) (USD > 125). More than 60% and 40% of the participants’ fathers and mothers had tertiary education, respectively. Approximately two-thirds of the participants’ fathers were involved in service, while most of their mothers were housewives.

**Table 1:** Characteristics of participants.

Variables	n (%)
*Gender*	
Male	200 (44.4)
Female	250 (55.6)
*Age (years)*	
≤20	43 (9.6)
21–25	382 (84.8)
>25	25 (5.6)
*Accommodation*	
University hall	321 (71.3)
With family/friends/mess	129 (28.7)
*Academic status*	
Undergraduate	388 (86.2)
Postgraduate	62 (13.8)
*Faculty*	
Science	264 (58.7)
Engineering	36 (8.0)
Business	44 (9.8)
Arts	106 (23.5)
*Marital status*	
Unmarried	422 (93.8)
Currently married	27 (6.0)
Divorced/separated/widowed	1 (0.2)
*Family residence*	
Rural	205 (45.6)
Urban	245 (54.4)
*Monthly family income (BDT)*	
≤15000 (USD ≤ 125)	145 (32.2)
>15000 (USD >125)	305 (67.8)
*Father’s education*	
No formal schooling	37 (8.2)
Primary	56 (12.4)
Secondary	77 (17.2)
Tertiary	280 (62.2)
*Father’s occupation*	
Service	299 (66.4)
Self-employed	151 (33.6)
*Mother’s education*	
No formal schooling	22 (4.9)
Primary	91 (20.2)
Secondary	128 (28.5)
Tertiary	209 (46.4)
*Mother’s occupation*	
Service	53 (11.8)
Housewife	397 (88.2)

Table 2 lists the mean scores for items assessing perceived stress among university students. As shown, the mean values ranged from 1.73 to 2.31, suggesting that the participants ‘sometimes’ experienced stress. Overall, the average score for PSS was 19.88 (SD = 4.67) out of 40 points, and none were noted to score minimum (0) or maximum (40) values. This indicated a 49.70% (19.88/40.00 × 100) PSS score, suggesting a moderate level of perceived stress among the participants. The goodness of fit indices indicated a good fit of the scale across multiple indicators (S2 Table). The Shapiro-Wilk test gave a p-value of 0.089 for PSS score, indicating that the data are normally distributed.

**Table 2:** Measurement items for the perceived stress scale among participants.

Questions (In the last month, how often have you …)	Mean	SD
been upset because of something that happened unexpectedly?	1.81	1.32
felt that you were unable to control the important things in your life?	2.04	1.13
felt nervous and stressed?	2.31	1.07
felt confident about your ability to handle your personal problems?	2.23	1.22
felt that things were going your way?	1.94	1.06
found that you could not cope with all the things that you had to do?	2.00	1.18
been able to control irritations in your life?	2.20	1.31
felt that you were on top of things?	1.73	1.17
been angered because of things that happened that were outside of your control?	1.97	1.27
felt difficulties were piling up so high that you could not overcome them?	1.86	1.23

SD: Standard deviation.

Of 450 students, more than half (57.3%) had low nutrition literacy, 47.6% had poor healthy eating behavior, and a very low proportion (2.7%) were involved in insufficient physical activity. Responses of the participants to each of the nutrition literacy and healthy eating-related questions are summarized in S3 and S4 Tables. Mean nutrition literacy was observed 19.14 ± 4.28 and the overall positive response rate for nutrition literacy was 59.8%. The average healthy eating behavior was found 20.89 ± 4.65 and the overall positive response rate for healthy eating behavior was 61.4% (20.89/34.0 × 100). The above findings indicate a moderate level of nutrition literacy and healthy eating behavior among the study subjects. NLS and HEBS exhibited acceptable fits across several key indicators (S2 Table). In Shapiro-Wilk test, p > 0.05 was noted for both NLS and HEBS, suggesting that the data did not deviate significantly from a normal distribution.

Table 3 shows the level of perceived stress among participants based on their level of nutrition literacy, physical activity and healthy eating behavior. Only significant diﬀerences were found for healthy eating behavior (P = 0.002). It was observed that the perceived stress level decreased as the adherence to healthy eating behavior was improved (20.61 ± 4.72 vs. 19.22 ± 4.53). No significant diﬀerences were found in their perceived stress level for nutrition literacy and physical activity.

**Table 3:** Relationship of perceived stress scores with nutrition literacy, healthy eating behavior, physical activity among participants.

Variable		Mean (SD)	t	Sig.
Nutrition literacy	Low	19.63 (4.52)	−1.332	0.184
	High	20.22 (4.86)		
Healthy eating behavior	Poor	20.61 (4.72)	3.180	0.002
	Good	19.22 (4.53)		
Physical activity	Insufficient	21.00 (3.39)	0.839	0.402
	Sufficient	19.85 (4.70)		

SD: Standard deviation.

**Fig 1:**
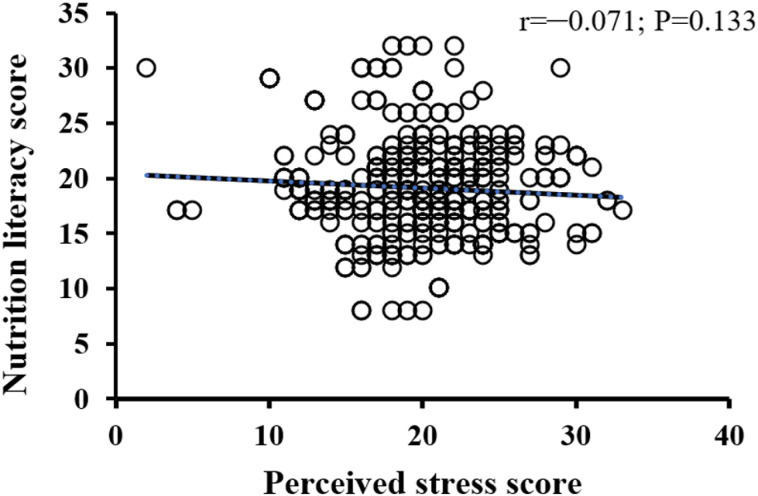
Correlation of perceived stress scores with nutrition literacyscores among participants.

**Fig 2:**
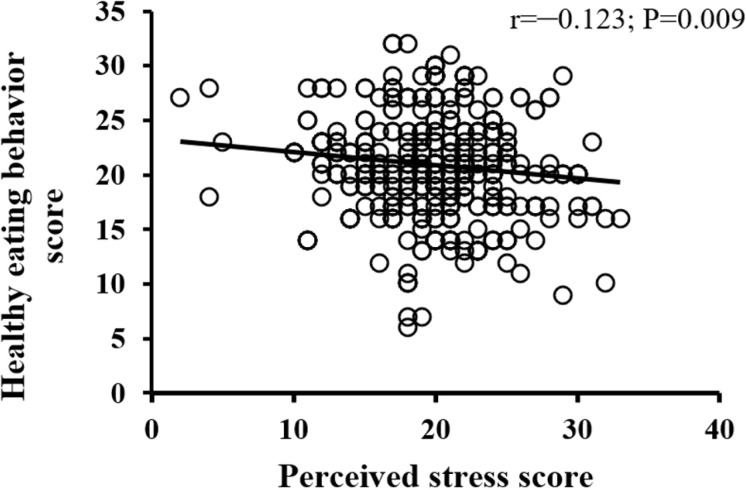
Correlation of perceived stress scores with healthy eating behavior scores among participants.

**Fig 3:**
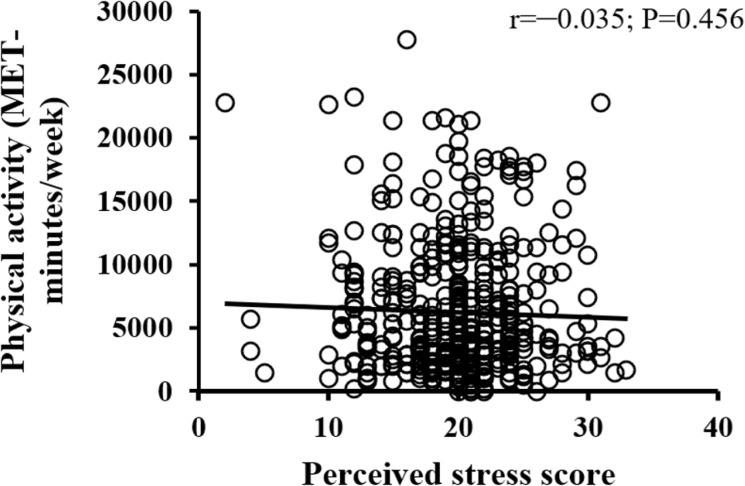
Correlation of perceived stress scores with physical activity among participants.

The bivariate correlations of students’ perceived stress with nutrition literacy, healthy eating behavior, and physical activity scores are illustrated in Figs 1–3. As shown in Figs 1–3, all variables under study had a negative association with perceived stress; however, only the healthy eating behavior score was statistically significant (r = –0.123; P = 0.009).

Table 4 presents the association of perceived stress with nutrition literacy, healthy eating behavior, and physical activity among students using the unadjusted and adjusted linear regression models. The data in the models passed the assumptions of the regression models (i.e., the residuals were approximately normally distributed and no specific pattern was observed in the scatterplot). Additionally, multicollinearity was assessed using Variance Inflation Factor (VIF) and, the values achieved were <5. Hence, the results were valid. The unadjusted model indicated that only healthy eating behavior was significantly negatively associated with perceived stress (*β* = –0.111, P = 0.023). However, after adjusting for the sociodemographic variables, nutrition literacy and healthy eating behavior were observed to be negatively associated with perceived stress. One unit increase in nutrition literacy and healthy eating behavior decreased the perceived stress score by 0.130 (P = 0.006) and 0.139 (P = 0.003) units, respectively, observed by the adjusted model (Table 4).

**Table 4:** Regression analysis for perceived stress explained by nutrition literacy, healthy eating behavior, and physical activity among participants.

Variable	Unadjusted model			Adjusted model^§^		
***β***	**95% CI**	**Sig.**	***β***	**95% CI**	**Sig.**
Nutrition literacy	−0.041	−0.095, 0.013	0.400	−0.130	−0.186, −0.092	0.006
Healthy eating behavior	−0.111	−0.206, −0.016	0.023	−0.139	−0.232, −0.028	0.003
Physical activity	−0.025	−0.126, 0.076	0.589	−0.039	−0.088, 0.010	0.395

*β*: Standardized coefficient. CI: Confidence interval. ^**§**^The regression model was adjusted for age, gender, accommodation, academic status, faculty of studying, marital status, family residence, monthly family income, father's education, father's occupation, mother's education, and mother's occupation.

## Discussion

The present study illustrates the relationship of perceived stress with nutrition literacy, healthy eating behavior, and physical activity among Bangladeshi university students. Results revealed a significant negative association of perceived stress with nutrition literacy and healthy eating behavior; however, no association was found with physical activity.

The present study showed a moderate level of perceived stress among the participants, with a mean PSS score of 19.88 ± 4.67. This result is similar to findings from previous research conducted in other countries such as Australia, China, Egypt, India, Saudi Arabia, and Taiwan [32–37]. Psychological stress is an inevitable aspect of human lives, affecting an individual’s physical well-being [38]. University students, in particular, are subjected to higher levels of stress due to several factors such as limited leisure time, extended teaching periods, rigorous examinations, and substantial workloads, making their stress levels exceed those of the general population [6]. This elevated stress levels observed among university students underscore the urgency of implementing measures to manage their stress. It is strongly advised to reduce academic pressures and offer counseling and academic assistance to support students effectively.

Moderate level of nutrition literacy and healthy eating practices were observed in this study which is in line with the previous study by Lai et al. [39] conducted among 412 university students in Taiwan and by Banna et al. [27] studied among 400 adult population of Dhaka and Chattogram district, Bangladesh. However, the plausible reason behind getting moderate nutrition literacy and healthy eating practice among students could be due to the availability and easy accessibility of National Dietary Guidelines for Bangladesh [40] that disseminate nutrition education among the adult population.

The prevalence of insufficient physical activity among the students was very low (2.7%) which is similar to the previous study by Al-Wardat et al. [14] reported that only 8.2% Jordanian university students had insufficient physical activity. NSTU provides dedicated sporting facilities and actively organizes sports activities for its students, creating an environment that encourages regular participation in physical activity. Additionally, the presence of on-campus residences and a rural campus setting may also facilitate easier access to open spaces and sports infrastructure, making it more convenient for students to engage in physical activities. On the other hand, university students may face various obstacles to engaging in physical activity, including insufficient time, inconvenience of exercising, lack of self-motivation, boredom with exercise, and lack of encouragement, support, or companionship from family and friends. Additionally, the absence of parks, sidewalks, bicycle trails, and other facilities could also be a barrier of doing sufficient physical activity [41,42].

The current study revealed that nutrition literacy was significantly negatively associated with perceived stress scores among the participants (Table 4). A pilot study by Jamshed et al. [9] reported that individuals with high nutrition literacy were less likely to experience depression and were three times more likely to possess self-esteem compared to those with low nutrition literacy. Additionally, low health and nutrition literacy levels were linked to poor health outcomes in both developed and developing countries, however, health/nutrition literacy was considered as a more critical predictor of mental health than other sociodemographic factors such as age, income, occupation, education, and race [43]. People with low health/nutrition literacy might find it challenging to discuss their health information or poor mental condition with friends and family loved due to feelings of discomfort and guilt. Conversely, high nutrition literacy may help individual to better cope with their mental stress [44].

Stress significantly influences eating behaviors, as demonstrated by the negative association between healthy eating behavior and perceived stress observed in this study (Table 4). This finding aligns with previous research, including a national survey conducted in the United States in 2007 revealed that almost half of the population reported feeling more stressed compared to five years prior, thus many began using consumption of unhealthy foods as a way to cope with their increased stress levels [45]. Shah et al. [46] reported that adolescents in Malaysian experiencing high stress levels were more prone to unhealthy eating behaviors, such as bingeing, snacking, and consuming high fat and sugar containing foods. Another study by Hsu and Raposa [47] observed that participants facing above-normal stress were more likely to eat specially unhealthy foods in response to difficult emotions on the same day. The physiological mechanism underlying this negative association between healthy eating behavior and stress involves multiple pathways. During periods of psychological or physical stress, the body releases glucocorticoids and insulin, which may trigger increased consumption of high-fat, high-sugar “comfort foods” while reducing intake of regular meals [48]. This stress-induced dietary pattern shift is particularly concerning because chronic consumption of unhealthy foods promotes visceral fat accumulation, which subsequently reduces the activity of the hypothalamic-pituitary-adrenal axis–a crucial component of the body's stress management system [48]. This creates a detrimental cycle where stress promotes unhealthy eating, which in turn compromises the body's ability to manage stress effectively. These findings have important implications for university health programs, suggesting that stress management interventions should incorporate nutrition education components, and conversely, nutrition programs should address stress management techniques.

The present study found no significant association between physical activity and perceived stress among university students (Table 4). This finding differs from previous studies that have reported stress-lowering effects of physical activity [15,16]. Several factors might explain this discrepancy. First, the high prevalence of sufficient physical activity in our sample might have created a ceiling effect, limiting variability and thus statistical power to detect associations. Second, the relationship between physical activity and stress might be moderated by other factors, such as motivation for exercise, enjoyment, or social aspects of the activity [49]. Our findings suggest that while physical activity is generally beneficial for health, its relationship with stress might be complex and context-dependent among university students in Bangladesh. Future studies should explore more nuanced measures of physical activity quality, motivation, and contextual factors to better understand this relationship.

The findings of this study have significant implications for promoting student well-being in academic settings. The negative association of perceived stress with nutrition literacy and healthy eating behavior suggests that universities should prioritize nutrition education programs and make healthy food options more accessible on campus. While physical activity did not show a direct link to stress reduction in this study, its overall health benefits still warrant inclusion in comprehensive wellness initiatives. These results call for a holistic approach to student health that combines dietary interventions with stress management strategies. University administrators should consider establishing wellness centers that offer nutrition counseling, mental health support, and physical activity programs. Policymakers may use these findings to advocate for national programs that enhance health literacy among young adults.

## Limitations

There are a few limitations of this study that are worth mentioning. Firstly, the study design itself is a limitation. The present study is descriptive and cross-sectional, which prevents the establishment of cause-effect relationships. Secondly, although a large number of subjects were included, the sample is not representative, which is noteworthy. Thirdly, being a university in a rural area, it had a rather uniform student. This homogeneity could limit the generalizability of the results to a more diverse student population. Lastly, potential biases may arise from using of self-reported data, as participants may overestimate or underestimate behaviors related to stress, nutrition literacy, eating habits, and physical activity due to social desirability bias or recall bias.

## Conclusion

The present study revealed a high prevalence of perceived stress, low nutrition literacy and poor healthy eating behavior among university students in Bangladesh; however, almost all did sufficient physical activity. The adjusted regression model highlighted a significant negative association of perceived stress with nutrition literacy and healthy eating behavior among participants. This pioneering study sheds light on the potential for targeted interventions that promote nutrition education and healthy lifestyle choices within academic environments. Future research should include diverse student populations across multiple institutions to enhance generalizability and explore the longitudinal impacts of nutrition and lifestyle factors on stress.

## Supporting information

S1 Original Questionnaire Original questionnaire. (DOCX)

S2 Survey Questionnaire Survey questionnaire. (DOCX)

Table S1 Results of the pilot study. (DOCX)

Table S2 The goodness of fit test parameters for the scales used. (DOCX)

Table S3 The status of nutrition literacy among participants. (DOCX)

Table S4 The status of healthy eating behavior among participants. (DOCX)
