# Supplementary material for: Association of stress with nutrition literacy, eating behavior, and physical activity: A cross-sectional study of university students in Bangladesh
Source: PLoS One. 2025 Jun 23;20(6):e0326269. doi: 10.1371/journal.pone.0326269 (PMC12184918; doi:10.1371/journal.pone.0326269)
Supplement: S1 Original Questionnaire — (DOCX) [file pone.0326269.s001.docx]

**প্রশ্নপত্র**

**সামাজিক জনসংখ্যাগত বৈশিষ্ট্য**

| **১. লিঙ্গ**  (ক) খারাপ  (খ) মহিলা | **২. বয়স......** |
| --- | --- |
| **৩. বৈবাহিক অবস্থা**  (ক) একক  (খ) বিবাহিত  (গ) তালাক | **৪. জীবনযাত্রার অবস্থা**  (ক) বিশ্ববিদ্যালয়েরবাসিন্দা  (খ) অনাবাসী |
| **৫.বর্তমান শিক্ষাবর্ষ**  (ক) অনার্স ১মবর্ষ  (খ) অনার্স ২য়বর্ষ  (গ) অনার্স ৩য়বর্ষ  (ঘ) অনার্স ৪র্থবর্ষ  (ঙ) মাস্টার্স | **৬.অনুষদ**  (ক) জীবনবিজ্ঞান  (খ) ইঞ্জিনিয়ারিং  (গ) ব্যবসায়িক অধ্যয়ন  (ঘ) কলা |
| **৭. আপনার পারিবারিক বাসস্থান কি?**  (ক) গ্রামীণ  (খ) শহুরে | **৮. পারিবারিক আয় (মাসিক)**  (ক) < ১০০০০  (খ) ১০০০০-১৫০০০  (গ) ১৫০০০এর বেশি |
| **৯. পিতার শিক্ষা**  (ক) প্রাথমিক  (খ) মাধ্যমিক  (গ) উচ্চ মাধ্যমিক বা উচ্চতর  (ঘ) প্রাতিষ্ঠানিক শিক্ষা নেই | **১০. মায়ের শিক্ষা**  (ক) প্রাথমি**ক**  (খ) মাধ্যমিক  (গ) উচ্চ মাধ্যমিক বা উচ্চতর  (ঘ) প্রাতিষ্ঠানিক শিক্ষা নেই |
| **১১. পিতার পেশা**  (ক) চাকরি  (খ) স্ব-নিযুক্ত | **১২. মায়ের পেশা**  (ক) চাকরি  (খ) গৃহিণী |

**Perceived Stress Scale**

প্রতিটি প্রশ্নের জন্য নিম্নলিখিত বিকল্পগুলি থেকে বেছে নিন: 0 - কখনই নয়, 1 - প্রায় কখনওই নয়, 2 - কখনও কখনও, 3 - মোটামুটি প্রায়ই, 4 - খুব প্রায়ই

| **প্রশ্ন** | **প্রতিক্রিয়া** |
| --- | --- |
| ১. গত মাসে, অপ্রত্যাশিতভাবে কোন কিছু ঘটে যাওয়ার কারণে আপনি কতবার মন খারাপ করেছেন? |  |
| ২. গত মাসে, আপনি কতবার অনুভব করেছেন যে আপনি আপনার জীবনের গুরুত্বপূর্ণ বিষয়গুলি নিয়ন্ত্রণ করতে অক্ষম ছিলেন? |  |
| ৩. গত মাসে, আপনি কতবার নার্ভাস এবং চাপ অনুভব করেছেন? |  |
| ৪. গত মাসে, আপনি আপনার ব্যক্তিগত সমস্যা সামলানোর ক্ষমতা সম্পর্কে কতবার আত্মবিশ্বাসী বোধ করেছেন? |  |
| ৫. গত মাসে, আপনি কতবার অনুভব করেছেন যে জিনিসগুলি আপনার ইচ্ছেমত চলছে? |  |
| ৬.গত মাসে, আপনি কতবার দেখেছেন যে আপনি সমস্ত জিনিসের সাথে মানিয়ে নিতে পারেননি? |  |
| ৭.গত মাসে, আপনি কতবার আপনার জীবনে বিরক্তি নিয়ন্ত্রণ করতে সক্ষম হয়েছেন? |  |
| ৮.গত মাসে, আপনি কতবার অনুভব করেছেন যে আপনি জিনিসগুলির শীর্ষে ছিলেন? |  |
| ৯.গত মাসে, আপনার নিয়ন্ত্রণের বাইরের ঘটনাগুলির কারণে আপনি কতবার রাগান্বিত হয়েছেন? |  |
| ১০.গত মাসে, আপনি কতবার অনুভব করেছেন যে অসুবিধাগুলি এত বেশি বেড়েছে যে আপনি সেগুলি কাটিয়ে উঠতে পারেননি? |  |

**Nutrition Literacy Scale**

পুষ্টি সাক্ষরতার স্কেল (আপনার প্রতিক্রিয়ার জন্য **√** চিহ্ন দিন)

| **প্রশ্ন** | খুব কঠিন | কঠিন | সহজ | খুব সহজ |
| --- | --- | --- | --- | --- |
| **পাওয়া** | | | | |
| ১.আমার জন্য, যখন পুষ্টি-সম্পর্কিত সমস্যা থাকে, তখন সঠিক তথ্য কোথায় পাওয়া যায় তা জানা |  |  |  |  |
| ২.আমার জন্য, যখন আমি সঠিক তথ্য কোথায় পেতে হবে তা জেনে সুস্থ-খাদ্য আচরণ শিখতে চাই |  |  |  |  |
| **বোঝা** | | | | |
| ৩.আমার জন্য, দৈনিক খাদ্য গাইডের বিষয়বস্তু বুঝতে সক্ষম হওয়া |  |  |  |  |
| ৪.আমার জন্য, বাংলাদেশের জন্য খাদ্যতালিকা নির্দেশিকাগুলির বিষয়বস্তু বুঝতে সক্ষম হওয়া |  |  |  |  |
| **বিশ্লেষণ করা** | | | | |
| ৫.আমার জন্য, খাদ্যের গ্রুপ এবং কাজগুলো আলাদা করার জন্য পুষ্টির দৃষ্টিকোণ থেকে খাবার বেছে নেওয়া |  |  |  |  |
| **মূল্যায়ন** | | | | |
| ৬.আমার জন্য, নেটওয়ার্কে পুষ্টির তথ্য সঠিক কিনা তা বিচার করা |  |  |  |  |
| **আবেদন করা** | | | | |
| ৭. আমার জন্য, এমন একটি পদ্ধতি বেছে নেওয়া যা আমার স্বাস্থ্যের চাহিদা পূরণ করে যখন স্বাস্থ্যকর খাবারের জন্য অনেক সুপারিশ থাকে |  |  |  |  |
| ৮.আমার জন্য, স্বাস্থ্যকর খাওয়ার জন্য দৈনন্দিন জীবনে সঠিক পুষ্টি তথ্য ব্যবহার করা হয় |  |  |  |  |

**Healthy Eating Behavior Scale**

স্বাস্থ্যকর খাবার আচরণ স্কেল (আপনার প্রতিক্রিয়ার জন্য ✓ চিহ্ন দিন)

| **খাওয়ার আচরণ এবং সম্পর্কিত বিষয়** | **খাবার ধরন** | | |
| --- | --- | --- | --- |
|  | **প্রতিদিন** | **মাঝে মাঝে** | **কখনোই না** |
| ১. খাদ্য পিরামিড এর ৬-৮ রকমের খাবার শ্রেণী থেকে বিভিন্ন খাবার গ্ৰহন |  |  |  |
| ২. পালিশহীন চাল ও আটা খাওয়া |  |  |  |
| ৩.ভিটামিন এ সমৃদ্ধ শাক-সবজি ও ফল খাওয়া |  |  |  |
| ৪.শাক-সবজি (পাতা যুক্ত ও পাতা বিহীন) খাওয়া |  |  |  |
| ৫.মাছ/ মাংস খাওয়া |  |  |  |
| ৬.ডাল খাওয়া |  |  |  |
| ৭.তেল ও চর্বি জাতীয় খাবার খাওয়া |  |  |  |
| ৮.মিস্টি জাতীয় খাবার খাওয়া |  |  |  |
| ৯.দুধ খাওয়া |  |  |  |
| ১০.টাটকা ও ভালোভাবে প্রস্তুত খাবার খাওয়া |  |  |  |
| ১১.অতিরিক্ত খাবার খাওয়া বাদ দেয়া |  |  |  |
| ১২.খাবার ভালো ভাবে চিবিয়ে খাওয়া |  |  |  |
| ১৩.খাবার আগে সর্বদা হাত ধোয়া |  |  |  |
| ১৪.প্রত্যেক সপ্তাহে নিজের ওজন মাপা |  |  |  |
| ১৫.নিয়মিত ব্যায়াম করা |  |  |  |
| ১৬.প্রতি বছর অন্তত একবার স্বাস্থ্য পরীক্ষা করা |  |  |  |
| ১৭.পর্যাপ্ত পরিমাণ বিশ্রাম নেওয়া ও ঘুমানো |  |  |  |

**Assessment of Physical Activity Level**

**শারীরিক কর্মক্ষমতার পরিমাণ যাচাই**

**ক. ভারী শারীরিক কার্যক্রম**

আপনি গত 7 দিনে অন্তত 10 মিনিট একবারে যে সমস্ত ভারী কার্যকলাপ করেছেন সেগুলি সম্পর্কে চিন্তা করুন। ভারী শারীরিক ক্রিয়াকলাপগুলি এমন ক্রিয়াকলাপগুলিকে বোঝায় যা কঠোর শারীরিক পরিশ্রম করে এবং আপনাকে স্বাভাবিকের চেয়ে অনেক বেশি শ্বাস নিতে বাধ্য করে।

P1. গত 7 দিনে, আপনি কত দিনে ভারী শারীরিক ক্রিয়াকলাপ করেছেন যেমন ভারী উত্তোলন, খনন, অ্যারোবিকস, বা দ্রুত সাইকেল চালানো?

............দিন/সপ্তাহ। *কোন জোরালো শারীরিক কার্যকলাপ নেই (প্রশ্ন P3 এ এড়িয়ে যান)

P2. আপনি সেই দিনগুলির মধ্যে ভারী শারীরিক ক্রিয়াকলাপ করার জন্য একদিনে সাধারণত কত সময় ব্যয় করেছিলেন?

প্রতিদিন .........ঘন্টা

প্রতিদিন ............মিনিট

জানি না/নিশ্চিত নয়

**খ. মাঝারি ধরনের শারীরিক কার্যক্রম**

আপনি গত 7 দিনে যে সমস্ত মাঝারি ধরনের কার্যকলাপ করেছেন সে সম্পর্কে চিন্তা করুন। শুধুমাত্র সেই শারীরিক ক্রিয়াকলাপগুলি সম্পর্কে চিন্তা করুন যা আপনি একবারে কমপক্ষে 10 মিনিটের জন্য করেছিলেন। মাঝারি ক্রিয়াকলাপগুলি বলতে এমন ক্রিয়াকলাপগুলিকে বোঝায় যা মাঝারি শারীরিক পরিশ্রম করে এবং আপনাকে স্বাভাবিকের চেয়ে কিছুটা কঠিন শ্বাস নিতে বাধ্য করে।

P3. গত 7 দিনে, আপনি কত দিনে হালকা ভার বহন, নিয়মিত গতিতে সাইকেল চালানো, বা ডাবল টেনিসের মতো মাঝারি শারীরিক ক্রিয়াকলাপ করেছেন? হাঁটা অন্তর্ভুক্ত করবেন না।

প্রতি সপ্তাহে...............দিন। “কোন মাঝারি শারীরিক কার্যকলাপ নেই (প্রশ্ন P5 এ এড়িয়ে যান)

P4. আপনি সেই দিনগুলির মধ্যে মাঝারি ধরনের শারীরিক ক্রিয়াকলাপ করার জন্য একদিনে সাধারণত কত সময় ব্যয় করেছিলেন?

প্রতিদিন .........ঘন্টা

প্রতিদিন ............মিনিট

জানি না/নিশ্চিত নয়

**গ. হাঁটা**

আপনি গত 7 দিনে হাঁটাহাঁটি করার সময় সম্পর্কে চিন্তা করুন। এর মধ্যে রয়েছে কর্মক্ষেত্রে এবং বাড়িতে, স্থান থেকে অন্য স্থানে ভ্রমণের জন্য হাঁটা, এবং আপনি শুধুমাত্র বিনোদন, খেলাধুলা, ব্যায়াম, বা অবসরের জন্য করেছেন এমন অন্য কোনো হাঁটা।

P5. গত 7 দিনে, আপনি কত দিনে অন্তত 10 মিনিট হাঁটলেন?

প্রতি সপ্তাহে ............দিন। “হাঁটা নেই (প্রশ্ন 7 এ এড়িয়ে যান)

P6. আপনি সাধারণত সেই দিনগুলির মধ্যে একটিতে কতটা সময় হাঁটতে ব্যয় করেছিলেন?

প্রতিদিন .........ঘন্টা

প্রতিদিন ............মিনিট

জানি না/নিশ্চিত নয়

**ঘ. সাপ্তাহিক ছুটির দিনে বসে থাকা**

শেষ প্রশ্নটি হল আপনি গত 7 দিনে সপ্তাহের দিনগুলিতে বসে থাকা সময় সম্পর্কে। কর্মক্ষেত্রে, বাড়িতে, কোর্সের কাজ করার সময় এবং অবসর সময়ে কাটানো সময় অন্তর্ভুক্ত করুন। এর মধ্যে একটি ডেস্কে বসে কাটানো, বন্ধুদের সাথে দেখা করা, পড়া, বা বসে থাকা বা টেলিভিশন দেখার জন্য শুয়ে থাকা সময় অন্তর্ভুক্ত থাকতে পারে।

P7. গত 7 দিনে, আপনি সপ্তাহের দিনে বসে কত সময় ব্যয় করেছেন?

প্রতিদিন .........ঘন্টা

প্রতিদিন ............মিনিট

জানি না/নিশ্চিত নয়
