# Supplementary material for: Association of stress with nutrition literacy, eating behavior, and physical activity: A cross-sectional study of university students in Bangladesh
Source: PLoS One. 2025 Jun 23;20(6):e0326269. doi: 10.1371/journal.pone.0326269 (PMC12184918; doi:10.1371/journal.pone.0326269)
Supplement: S2 Survey Questionnaire — (DOCX) [file pone.0326269.s002.docx]

**Questionnaire**

**Sociodemographic characteristics**

| **1. Gender**  (a) Male  (b) Female | **2. Age ……………** |
| --- | --- |
| **3. Marital status**  (a) Unmarried  (b) Currently married  (c) Divorced/separated/widowed | **4. Accommodation**  (a) University hall  (b) With family/friends/mess |
| **5. Academic status**   1. Honors 1st year 2. Honors 2^nd^ year 3. Honors 3^rd^ year 4. Honors 4^th^ year   (e) Masters | **6. Faculty**   1. Life science 2. Engineering 3. Business study   (d) Arts |
| **7. What is your family residence?**  (a) Rural  (b) Urban | **8. Family income (monthly)**  (a) <10000  (b) 10000-15000  (c) More than15000 |
| **9. Father’s education**  (a) Primary  (b) Secondary  (c) Tertiary  (d) No formal schooling | **10. Father’s education**  (a) Primary  (b) Secondary  (c) Tertiary  (d) No formal schooling |
| **11. Father’s occupation**  (a) Service  (b) Self-employed | **12. Mother’s occupation**  (a) Service  (b) Housewife |

**Perceived Stress Scale**

For each question choose from the following alternatives: **0 - never, 1 - almost never, 2 – sometimes, 3 - fairly often, 4 - very often**

| **Questions** | **Response** |
| --- | --- |
| l. In the last month, how often have you been upset because of something that happened unexpectedly? |  |
| 2. In the last month, how often have you felt that you were unable to control the important things in your life? |  |
| 3. In the last month, how often have you felt nervous and stressed? |  |
| 4. In the last month, how often have you felt confident about your ability to handle your personal problems? |  |
| 5. In the last month, how often have you felt that things were going your way? |  |
| 6. In the last month, how often have you found that you could not cope with all the things that you had to do? |  |
| 7. In the last month, how often have you been able to control irritations in your life? |  |
| 8. In the last month, how often have you felt that you were on top of things? |  |
| 9. In the last month, how often have you been angered because of things that happened that were outside of your control? |  |
| 10. In the last month, how often have you felt difficulties were piling up so high that you could not overcome them? |  |

**Nutrition literacy scale (Give √ marks for your response)**

| **Questions** | **Very difficult** | **Difficult** | **Easy** | **Very easy** |
| --- | --- | --- | --- | --- |
| **Obtain** | | | | |
| 1. For me, when there are nutrition-related issues, knowing where to find the right information is |  |  |  |  |
| 2. For me, when I want to learn healthy-eating behaviors knowing where to find the right information is |  |  |  |  |
| **Understand** | | | | |
| 3. For me, being able to understand the contents of the Daily Food Guide is … |  |  |  |  |
| 4. For me, being able to understand the contents of the Dietary Guidelines for Bangladesh is … |  |  |  |  |
| **Analyze** | | | | |
| 5. For me, choosing foods from the nutritional point of view to distinguish food groups and functions is |  |  |  |  |
| **Appraise** | | | | |
| 6. For me, judging whether the nutrition information on the network is correct or not is |  |  |  |  |
| **Apply** | | | | |
| 7. For me, choosing a method that meets my health need when there are many recommendations for healthy diets is |  |  |  |  |
| 8. For me, using the right nutrition information in daily life for healthy eating is |  |  |  |  |

**Healthy eating behavior scale (Give √ marks for your response)**

| **Eating behavior and related issues** | **Frequency of eating** | | |
| --- | --- | --- | --- |
|  | **Regular** | **Occasionally** | **Never** |
| 1. Eat a variety of food from 6-8 food groups of food pyramid |  |  |  |
| 2. Eat unpolished rice, wheat |  |  |  |
| 3. Eat vegetables vit A rich fruits |  |  |  |
| 4. Eat vegetables (leafy & non leafy) |  |  |  |
| 5. Eat fish/meat |  |  |  |
| 6. Eat Pulses |  |  |  |
| 7. Eat foods containing fat and oils |  |  |  |
| 1. Eat sweetened foods |  |  |  |
| 1. Drink milk |  |  |  |
| 1. Eat fresh, well-prepared foods |  |  |  |
| 1. Avoid overeating |  |  |  |
| 1. Eat food with proper chewing |  |  |  |
| 1. Always wash hands before meals |  |  |  |
| 1. Have your body weight measured weekly |  |  |  |
| 1. Perform exercise |  |  |  |
| 1. Undertake clinical check-up at least once a year |  |  |  |
| 1. Take enough rest and sleep |  |  |  |

**Assessment of physical activity level**

**A*.* Vigorous physical activity**

Think about all **the vigorous activities** that you did in the **last 7 days** at least **10 minutes** at **a time**. *Vigorous physical activities refer to activities that take hard physical effort and make you breathe much harder than normal.*

**P1.** During the last 7 days, on how many days did you do vigorous physical activities like heavy lifting, digging, aerobics, or fast bicycling?

_____ days/ week. *No vigorous physical activities (Skip to question 3)

**P2.** How much time did you usually spend doing vigorous physical activities on one of those days?

_____ hours per day

_____ minutes per day

Don’t know/Not sure

**B*.* Moderate physical activity**

Think about all the moderate activities that you did in the **last 7 days**. Think only about those physical activities that you did for **at least 10 minutes** at **a time**. *Moderate activities refer to activities that take moderate physical effort and make you breathe somewhat harder than normal.*

**P3.** During the last 7 days, on how many days did you do moderate physical activities like carrying light loads, bicycling at a regular pace, or doubles tennis? Do not include walking.

_____ days per week**. ^*^**No moderate physical activities (Skip to question 5)

**P4.** How much time did you usually spend doing moderate physical activities on one of those days?

_____ hours per day

_____ minutes per day

Don’t know/Not sure

**C. Walking**

Think about the time you spent walking in the last 7 days. *This includes at work and at home, walking to travel from place to place, and any other walking that you have done solely for recreation, sport, exercise, or leisure.*

**P5.** During the last 7 days, on how many days did you walk for at least 10 minutes at a time?

_____ days per week. ^*^No walking (Skip to question 7)

**P6.** How much time did you usually spend walking on one of those days?

_____ hours per day

_____ minutes per day

Don’t know/Not sure

**D. Sitting on weekdays**

The last question is about the time you spent sitting on weekdays during the last 7 days. *Include time spent at work, at home, while doing course work and during leisure time. This may include time spent sitting at a desk, visiting friends, reading, or sitting or lying down to watch television.*

**P7.** During the last 7 days, how much time did you spend sitting on a week day?

_____ hours per day

_____ minutes per day

Don’t know/Not sure
