# Supplementary material for: Association of stress with nutrition literacy, eating behavior, and physical activity: A cross-sectional study of university students in Bangladesh
Source: PLoS One. 2025 Jun 23;20(6):e0326269. doi: 10.1371/journal.pone.0326269 (PMC12184918; doi:10.1371/journal.pone.0326269)
Supplement: Table S1 — (DOCX) [file pone.0326269.s003.docx]

**Table S1.** Results of the pilot study.

|  | **Findings** |
| --- | --- |
| Descriptive statistics | Total participants: 30   - Male: 16 (53.3%) - Female: 14 (46.7%)   Age range: 19-25 years  Average completion time: 13.5 minutes |
| Scale reliability | Perceived stress scale (PSS-10)   - Cronbach's alpha: 0.722   Nutrition literacy scale (NLS)   - Cronbach's alpha: 0.797   Healthy eating behavior scale (HEBS)   - Cronbach's alpha: 0.703 |
| Modifications | Nutrition literacy scale   - Rephrased two items in the Nutrition Literacy Scale to improve comprehension   Healthy eating behavior scale   - Added local examples of foods |
