# Supplementary material for: Association of stress with nutrition literacy, eating behavior, and physical activity: A cross-sectional study of university students in Bangladesh
Source: PLoS One. 2025 Jun 23;20(6):e0326269. doi: 10.1371/journal.pone.0326269 (PMC12184918; doi:10.1371/journal.pone.0326269)
Supplement: Table S2 — (DOCX) [file pone.0326269.s004.docx]

**Table S2.** The goodness of fit test parameters for the scales used.

| **Fit indices** | **Accepted value** | **Scale value** | | | **Comment** |
| --- | --- | --- | --- | --- | --- |
|  |  | **PSS** | **NLS** | **HEBS** |  |
| Comparative fit index (CFI) | ≥0.95 | 0.986 | 0.996 | 0.959 | Acceptable fit |
| Tucker-Lewis index (TLI) | ≥0.95 | 0.964 | 0.987 | 0.918 | Acceptable fit |
| Root mean square error of approximation (RMSEA) | ≤0.06 | 0.043 | 0.032 | 0.040 | Acceptable fit |
| Standardized root mean square residual (SRMR) | <0.08 | 0.029 | 0.018 | 0.039 | Acceptable fit |
| Chi-square to degrees of freedom ratio (χ^2^/*df*) | ≤2.00 | 1.833 | 1.471 | 1.735 | Acceptable fit |

PSS: Perceived stress scale, NLS: Nutrition literacy scale, HEBS: Healthy eating behavior scale.
