# Supplementary material for: Association of stress with nutrition literacy, eating behavior, and physical activity: A cross-sectional study of university students in Bangladesh
Source: PLoS One. 2025 Jun 23;20(6):e0326269. doi: 10.1371/journal.pone.0326269 (PMC12184918; doi:10.1371/journal.pone.0326269)
Supplement: Table S3 — (DOCX) [file pone.0326269.s005.docx]

**Table S3.** The status of nutrition literacy among participants.

| **Item** | **Very difficult (%)** | **Difficult (%)** | **Easy (%)** | **Very easy (%)** | **Mean (SD)** |
| --- | --- | --- | --- | --- | --- |
| *Obtain* |  |  |  |  |  |
| 1. For me, when there are nutrition-related issues, knowing where to find the right information is … | 74 (16.4) | 189 (42.0) | 134 (29.8) | 53 (11.8) | 2.37 (0.89) |
| 1. For me, when I want to learn healthy-eating behaviors knowing where to find the right information is … | 27 (6.0) | 189 (42.0) | 187 (41.6) | 47 (10.4) | 2.56 (0.76) |
|  |  |  |  |  |  |
| *Understand* |  |  |  |  |  |
| 1. For me, being able to understand the contents of the Daily Food Guide is … | 49 (10.9) | 166 (36.8) | 192 (42.7) | 43 (9.6) | 2.51 (0.81) |
| 1. For me, being able to understand the contents of the Dietary Guidelines for Bangladesh is … | 58 (12.9) | 234 (52.0) | 134 (29.8) | 24 (5.3) | 2.28 (0.75) |
|  |  |  |  |  |  |
| *Analyze* |  |  |  |  |  |
| 1. For me, choosing foods from the nutritional point of view to distinguish food groups and functions is … | 66 (14.7) | 220 (48.9) | 138 (30.6) | 26 (5.8) | 2.28 (0.78) |
|  |  |  |  |  |  |
| *Appraise* |  |  |  |  |  |
| 1. For me, judging whether the nutrition information on the network is correct or not is … | 66 (14.7) | 184 (40.8) | 169 (37.6) | 31 (6.9) | 2.37 (0.82) |
|  |  |  |  |  |  |
| *Apply* |  |  |  |  |  |
| 1. For me, choosing a method that meets my health need when there are many recommendations for healthy diets is … | 62 ((13.8) | 216 (48.0) | 138 (30.6) | 34 (7.6) | 2.32 (0.80) |
| 1. For me, using the right nutrition information in daily life for healthy eating is … | 43 (9.6) | 193 (42.8) | 180 (40.0) | 34 (7.6) | 2.46 (0.77) |

SD: Standard deviation.
