# Supplementary material for: Association of stress with nutrition literacy, eating behavior, and physical activity: A cross-sectional study of university students in Bangladesh
Source: PLoS One. 2025 Jun 23;20(6):e0326269. doi: 10.1371/journal.pone.0326269 (PMC12184918; doi:10.1371/journal.pone.0326269)
Supplement: Table S4 — (DOCX) [file pone.0326269.s006.docx]

**Table S4.** The status of healthy eating behavior among participants.

| **Item** | **Regular (%)** | **Occasionally (%)** | **Never (%)** | **Mean (SD)** |
| --- | --- | --- | --- | --- |
| Eat a variety of food from 6-8 food groups of food pyramid | 148 (32.9) | 281 (62.4) | 21 (4.7) | 1.28 (0.55) |
| Eat unpolished rice, wheat | 148 (32.9) | 201 (44.7) | 101 (22.4) | 1.10 (0.74) |
| Eat vegetables vit A rich fruits | 148 (32.9) | 260 (57.8) | 42 (9.3) | 1.24 (0.61) |
| Eat vegetables (leafy & non leafy) | 222 (49.3) | 195 (43.4) | 33 (7.3) | 1.42 (0.63) |
| Eat fish/meat | 266 (59.1) | 159 (35.3) | 25 (5.6) | 1.54 (0.60) |
| Eat Pulses | 158 (35.1) | 210 (46.7) | 82 (18.2) | 1.17 (0.71) |
| Eat foods containing fat and oils | 236 (52.4) | 168 (37.4) | 46 (10.2) | 1.42 (0.67) |
| Eat sweetened foods | 144 (32.0) | 250 (55.6) | 56 (12.4) | 1.20 (0.64) |
| Drink milk | 122 (27.1) | 247 (54.9) | 81 (18.0) | 1.09 (0.67) |
| Eat fresh, well-prepared foods | 169 (37.6) | 220 (48.9) | 61 (13.6) | 1.24 (0.67) |
| Avoid overeating | 237 (52.7) | 168 (37.3) | 45 (10.0) | 1.43 (0.67) |
| Eat food with proper chewing | 235 (52.2) | 152 (33.8) | 63 (14.0) | 1.38 (0.72) |
| Always wash hands before meals | 237 (52.7) | 132 (29.3) | 81 (18.0) | 1.35 (0.77) |
| Have your body weight measured weekly | 179 (29.8) | 164 (36.4) | 107 (23.8) | 1.16 (0.78) |
| Perform exercise | 157 (34.9) | 198 (44.0) | 95 (21.1) | 1.14 (0.74) |
| Undertake clinical check-up at least once a year | 155 (34.4) | 184 (40.9) | 111 (24.7) | 1.10 (0.76) |
| Take enough rest and sleep | 270 (60.0) | 131 (29.1) | 49 (10.9) | 1.49 (0.69) |

SD: Standard deviation.
